# Long-term effects of blood pressure 130–139/80–89 mmHg on all-cause and cardiovascular mortality among Chinese adults with different glucose metabolism

**DOI:** 10.1186/s12933-023-02088-9

**Published:** 2023-12-21

**Authors:** Chang-Sheng Sheng, Haiyan Wang, Yanjun Liu, Yanyun Li, TianZhiChao Hou, Miaoyan Qiu, Yao Lu, Siming Sun, Junhan Yang, Xiaomin Song, Guang Ning, Jingyan Tian

**Affiliations:** 1 Department of Cardiovascular Medicine, Center for Epidemiological Studies and Clinical Trials, Center for Vascular Evaluation, Shanghai Key Lab of Hypertension, Shanghai Institute of Hypertension, Ruijin Hospital, Shanghai Jiaotong University School of Medicine, Shanghai, China; 2 Pingliang Community Health Service Center, Yangpu District, Shanghai, China; 3 College of Food Science and Engineering, Ocean University of China, Qingdao, Shandong Province China; 4 Division of Chronic Non-Communicable Disease and Injury, Shanghai municipal center for disease control and prevention, Shanghai, 200336 China; 5 Department of Endocrine and Metabolic Diseases, Shanghai Institute of Endocrine and Metabolic Diseases, Ruijin Hospital, Shanghai Jiao Tong University School of Medicine, Shanghai, China; 6 Shanghai National Clinical Research Center for Metabolic Diseases, Key Laboratory for Endocrine and Metabolic Diseases of the National Health Commission of the PR China, Shanghai Key Laboratory for Endocrine Tumor, Ruijin Hospital, Shanghai Jiao Tong University School of Medicine, Shanghai, China; 7 Department of Endocrinology, Xinhua Hospital, Shanghai JiaoTong University School of Medicine, Shanghai, 200092 China; 8 Department of Endocrinology and metabolism, Yangpu Hospital, Tongji University School of Medicine, Shanghai, China; 9 Acute infectious disease control Department, Shanghai Hongkou Center for Disease Control and Prevention, Shanghai, China

**Keywords:** High Blood Pressure, All-cause mortality, Cardiovascular mortality, Glucose metabolism, Chinese

## Abstract

**Background:**

This study aimed to investigate the risks of all-cause and cardiovascular mortality associated with blood pressure (BP) levels of 130–139/80–89 mmHg in Chinese adults with different glucose metabolism, during a long-term follow-up of over 20 years.

**Methods:**

A prospective population-based cohort of 2,132 adults in Shanghai was established in 2002 and followed for 21 years. The association between BP categories and mortality was assessed, and the risk was further analyzed using multiple Cox regression analysis by combining BP and blood glucose categories.

**Results:**

The final analysis included 2,004 participants, with 397 all-cause and 166 cardiovascular mortality. The incidence of all-cause and cardiovascular mortality per 1,000 person-years for different BP categories were as follows: BP < 130/80 mmHg (4.5 and 1.3), 130–139/80–89 mmHg (7.7 and 2.9), and ≥ 140/90 mmHg or treated groups (19.9 and 8.7), respectively. After adjusting for age, sex, and other factors, BP ≥ 140/90 mmHg was significantly associated with a higher risk of mortality across different blood glucose categories. However, using BP < 130/80 mmHg and normoglycemia as the reference, a BP of 130–139/80–89 mmHg was significantly associated with higher risks of all-cause (hazard ratio 3.30 [95% confidence interval 1.48–7.38], *P* < 0.01) and cardiovascular mortality (9.60 [1.93–47.7], *P* < 0.01) in diabetes, but not in those with normoglycemia or prediabetes.

**Conclusions:**

BP of 130–139/80–89 mmHg may lead to a significantly higher risk of all-cause and cardiovascular mortality in Chinese adults with diabetes, but not in those with normoglycemia or prediabetes. This suggests that the targeted BP for people with diabetes should be < 130–139/80–89 mmHg.

## Background

Observational studies have consistently demonstrated a strong correlation between elevated blood pressure (BP) and the development of cardiovascular disease (CVD) [1, 2]. These studies have provided valuable insights into the relationship between BP and CVD risk, highlighting the importance of maintaining optimal BP levels for long-term cardiovascular health. A comprehensive meta-analysis of 61 prospective studies further supports these findings by revealing a clear dose-response relationship between BP levels and CVD risk [1]. The analysis showed that individuals with systolic blood pressure (SBP) levels below 115 mmHg and diastolic blood pressure (DBP) levels below 75 mmHg had the lowest risk of CVD. As BP levels increased beyond these thresholds, the risk of developing CVD increased logarithmically.

In recent years, several hypertension guidelines have been released, [3–6] and there is growing concern about blood pressure levels of 130–139/80–89 mmHg. According to the 2017 AHA/ACC guidelines, [3] this range is classified as stage 1 hypertension and requires therapy initiation if accompanied by clinical atherosclerotic cardiovascular disease or an estimated 10-year CVD risk of ≥ 10%. However, the 2018 Chinese hypertension guidelines, [4] 2020 ISH guidelines, [5] and 2023 European guidelines [6] consider 130–139/80–89 mmHg as “prehypertension” or “high normal” or an overlap between the two. Nevertheless, the recent guidelines all recommend a target blood pressure of 130/80 mmHg for hypertensive patients, either alone or in combination with other cardiovascular risk factors [6, 7].

Hypertension often coexists with other cardiovascular risk factors, such as impaired glucose metabolism. There is a higher prevalence of hypertension among individuals with type 2 diabetes (T2D), and vice versa, which increases the risk of CVD events [8]. Accumulated evidence strongly suggests that reducing BP in individuals with T2D can have a substantial positive impact on cardiovascular outcomes [9, 10]. Guidelines from organizations such as ACC/AHA,[3] AACE/ACE,[11] ESC/EASD,[12] and ADA [13] recommend a BP target of < 130/80 mmHg for patients with T2D. The ESC/EASD guidelines also provide recommendations for managing BP in individuals with diabetes and prediabetes [12]. However, there are still some studies with varying conclusions regarding BP targets for diabetes and prediabetes. Recent meta-analyses demonstrated that a BP target range of > 130/80 to < 140/90 mmHg may be optimal for patients ≥ 65 years with T2D, but specific targets may need to be individualized based on patients’ unique circumstances [14]. Reducing diastolic BP, particularly below 60 mmHg, is associated with increased risk of all-cause mortality [15]. Moreover, at early stage of diabetes, a slightly elevated systolic BP (140–150 mmHg) is transiently associated with better beta-cell function in T2D patients with HbA1c ≥ 10% but not in those with HbA1c < 10% [16]. This is a significant gap in our understanding, considering the increasing prevalence of prediabetes and its potential implications for both clinical management and public health.

In our previous study, we investigated the risk of CVD events in Chinese adults with a BP range of 130–139/80–89 mmHg and varying glucose metabolism [17]. By conducting a follow-up study for over 20 years, we collected data on vital status and cause of death, allowing us to investigate the long-term associations between blood pressure levels of 130–139/80–89 mmHg and mortality from all causes and cardiovascular diseases among Chinese adults with different glucose metabolism. We hypothesize that the BP range of 130–139/80–89 mmHg in patients with diabetes may result in a significantly increased risk of mortality, similar to the risk observed for CVD events in our previous study.

## Methods

### Study design and participants

The prospective population-based cohort of 2,132 adults in Shanghai was established in 2002, and the study design had been described previously [17–21]. The first examination was conducted from November 2002 to January 2003. At baseline, all participants underwent physical examination and were interviewed with standardized questionnaires. Data were collected on physician diagnosed diabetes and hypertension, family history of diabetes, educational background, cigarette smoking, alcohol consumption, and currently used medications for hypertension and diabetes. An oral glucose tolerance test and serum lipid profile were assayed in all participants. Till now, 4 times follow-up were conducted in 2004, 2013–2014, 2018–2020, and 2022–2023, respectively. All participants at follow-up were interviewed with the same standardized questionnaires. At last, after excluding 128 participants lost to follow-up over four follow-up periods, a total of 2,004 participants were followed among 21 years’ follow-up, and were included in the final analysis, including 397 all-cause mortality and 166 cardiovascular mortality. Cardiovascular mortality included deaths attributable to stroke, myocardial infarction, and other cardiovascular diseases. Information on all-cause mortality and cardiovascular mortality were firstly obtained during this follow-up from the family members of the deceased people, and then were confirmed from the official death certificates of the district. This study was approved by Ruijin Hospital ethics committee and written informed consent was obtained from each participant.

### Measurements and definition of variables

BP was measured three times using a mercury sphygmomanometer while the participant was in a sitting position after a 5-minute rest. SBP and DBP were determined as the average of the last two of the three measurements. BP categories were defined as follows: <130/80 mmHg, 130–139/80–89 mmHg, and ≥ 140/90 mmHg or treated. Plasma glucose levels were measured during a 75-g oral glucose tolerance test. Diabetes was defined by a fasting plasma glucose (FPG) level ≥ 7.0 mmol/L and/or a 2-hour post-challenge glucose (2hPG) level ≥ 11.1 mmol/L, a previous physician diagnosis of diabetes, or the use of antidiabetic medication at baseline. Prediabetes was defined as FPG between 5.6 and 7.0 mmol/L and/or 2hPG between 7.8 and 11.1 mmol/L [22]. Normoglycemia was defined by FPG < 5.6 mmol/L and 2hPG < 7.8 mmol/L. Triglycerides (TG), total serum cholesterol (TC), HDL, and LDL levels were measured enzymatically.

Anthropometric measurements were conducted by 20 trained nurses or clinical postgraduates. Smoking and alcohol consumption, educational background, diet, and physical activity were acquired through well designed questionnaires. Family history of diabetes was defined as at least one first-degree relative or grandparents with diabetes. Smoking and drinking status were classified as currently, formerly, and never consumed. Physical activity was calculated as the product of the duration and frequency of each activity (in hours per day) weighted by an estimate of the metabolic equivalent of that activity. Ten-year atherosclerotic CVD (ASCVD) risk was estimated using prediction equations validated by the Prediction for ASCVD Risk in China project, [23] which evolved from The Framingham Heart Study, but is more suitable for Chinese people. In the prediction equations, the following major risk factors are included: age, treated or untreated SBP, total cholesterol, HDL-C, current smoking, and diabetes mellitus. Additionally, for men, 4 additional variables—waist circumference, geographic region, urbanization, and family history of ASCVD—have been added to the equation. For women, 2 additional variables—waist circumference, and geographic region—have been included [23].

### Statistical analysis

We used SAS 9.4 software for database management and statistical analysis. Data are expressed as mean ± SD, %, geometric mean with 95% limits, or hazard ratios (HRs) with 95% CIs. All statistical tests were two-sided, with *P* < 0.05 considered significant. Serum TG values were log-transformed before analysis because of skewed distribution. Multiple Cox regression analysis was performed to compute HRs (95% CIs). The log-rank test was used to compare the cumulative incidence of mortality between groups, with the Kaplan Meier survival function used to show the time to events. Proportionality assumptions for the Cox models were assessed by diagnostic plots of the scaled Schoenfeld residuals and log-minus-log survival plots. Substantial deviations from proportionality were not observed. We considered individuals with BP < 130/80 mmHg (or with normoglycemia, if considered) as the reference group. Dummy variables were used to compute HRs (95% CIs) for each subgroup against the reference group.

## Results

### Baseline characteristics

The median of follow-up time was 16.5 (interquartile range, 10.8 to 20.2) years. The mean values of anthropometric data and metabolic variables by BP category at baseline are presented in Table 1. Participants with a BP of 130–139/80–89 mmHg and ≥ 140/90 mmHg or treated were more likely to be male and to have more cigarette or alcohol consumption.

**Table 1:** Characteristics of the study participants, according to the BP categories at baseline

Variables	*Overall* *(N = 2004)*	BP categories (mmHg)			*P Value* ^*&*^
		***< 130/80*** ***(N = 407)***	***130–139/80–89*** ***(N = 520)***	≥ ***140/90 or treated*** ***(N = 1077)***	
SBP(mmHg)	130.7 ± 19.5	109.4 ± 9.2	122.0 ± 8.3	143.0 ± 16.8	**< 0.0001**
DBP(mmHg)	83.6 ± 10.9	69.8 ± 5.2	82.0 ± 3.5	89.6 ± 9.7	**< 0.0001**
Age (years)	54.6 ± 13.1	47.3 ± 14.2	51.1 ± 12.8	59.1 ± 10.8	**< 0.0001**
Male prevalence, %	39.9	29.7	41.2	43.2	**< 0.0001**
FPG(mmol/l)	6.0 ± 1.8	5.6 ± 1.5	5.7 ± 1.5	6.3 ± 2.0	**< 0.0001**
2hPG(mmol/l)	6.0 ± 2.8	5.3 ± 2.4	5.5 ± 2.2	6.5 ± 3.1	**< 0.0001**
LDL(mmol/l)	1.4 ± 0.4	1.5 ± 0.4	1.5 ± 0.5	1.4 ± 0.4	**< 0.0001**
HDL(mmol/l)	2.8 ± 0.8	2.6 ± 0.7	2.7 ± 0.8	3.0 ± 0.8	**< 0.0001**
TG(mmol/l)#	1.29 (1.26–1.33)	1.02 (0.97–1.07)	1.20 (1.14–1.26)	1.46 (1.41–1.51)	**< 0.0001**
TC(mmol/l)	4.9 ± 1.0	4.7 ± 0.9	4.8 ± 0.9	5.1 ± 1.0	**< 0.0001**
BMI (Kg/m^2^)	25.1 ± 3.5	23.3 ± 3.3	24.4 ± 3.2	26.0 ± 3.4	**< 0.0001**
Waist circumference (cm)	80.1 ± 9.5	75.2 ± 9.0	78.3 ± 8.2	82.9 ± 9.4	**< 0.0001**
Waist:hip ratio	0.8 ± 0.1	0.8 ± 0.1	0.8 ± 0.1	0.8 ± 0.1	**< 0.0001**
Family history of diabetes%	18.6	24.1	18.1	16.7	**0.005**
Alcohol use %					**< 0.0001**
Currently and Former	15.0	7.4	13.7	18.6	
Never	86.0	92.6	86.3	81.4	
Smoking status %					**0.002**
Current and Former	23.1	16.8	26.0	24.0	
Never	76.9	823.2	74.0	76.0	
Physical activity %					**0.42**
Inactive	38.3	35.0	40.8	38.3	
Medium	34.9	35.7	34.8	34.6	
Active	26.8	29.3	24.4	27.1	
ASCVD score (%)*	3.67 (1.38–8.08)	0.91 (0.47–2.10)	2.00 (1.04–3.97)	6.73 (3.48–11.03)	**< 0.0001**
ASCVD score ≥ 10%, %	18.0	2.3	3.8	30.3	**< 0.0001**

Abbreviation: SBP: systolic blood pressure; DBP: diastolic blood pressure; FPG: fasting plasma glucose; 2hPG, 2-hour post-challenge glucose; LDL: low-density lipoprotein; HDL: high density lipoprotein. TG: Triglycerides; TC: total serum cholesterol; BMI: body mass index

Results are given mean ± SD or n (%) or geometric mean (95% Limits)

^#^Serum TG values were log-transformed before analysis because of skewed distribution. *ASCVD score (%) was expressed as median (lower and upper quartiles). ^&^Means and proportions were compared across different BP categories using one-way ANOVA and Fisher’s exact test, respectively

Compared with those with BP < 130/80 mmHg, age, FPG, 2hPG, LDL, TC, TG, BMI, waist circumference, and waist-to-hip ratio were higher among participants with BP 130–139/80–89 mmHg and the highest among participants with BP ≥ 140/90 mmHg or treated. Inverse relationship was observed between the prevalence of diabetes family history and BP categories. No significant difference was found in physical activity among these three groups. Moreover, we compared the baseline characteristics between participants who attended follow-up with those lost to follow-up and found no significant difference in glucose levels, blood lipid profile, BP, and any other factors between the two groups (data not shown).

Among the 1077 participants with hypertension at baseline, 379 (35.2%) were currently taking antihypertensive medications. Among the 309 participants with T2D, 181 (58.6%) were currently taking antidiabetic medications. Among those taking antidiabetic medications, 55.2% were using metformin (17% in combination with insulin, 37% in combination with sulfonylurea, and 10% in combination with glucosidase inhibitors), 23.3% were using insulin, 43.1% were using sulfonylurea, 21.5% were using glucosidase inhibitors, and 42.5% were using two or more drugs.

### BP categories and all-cause and cardiovascular mortality

**Fig. 1:**
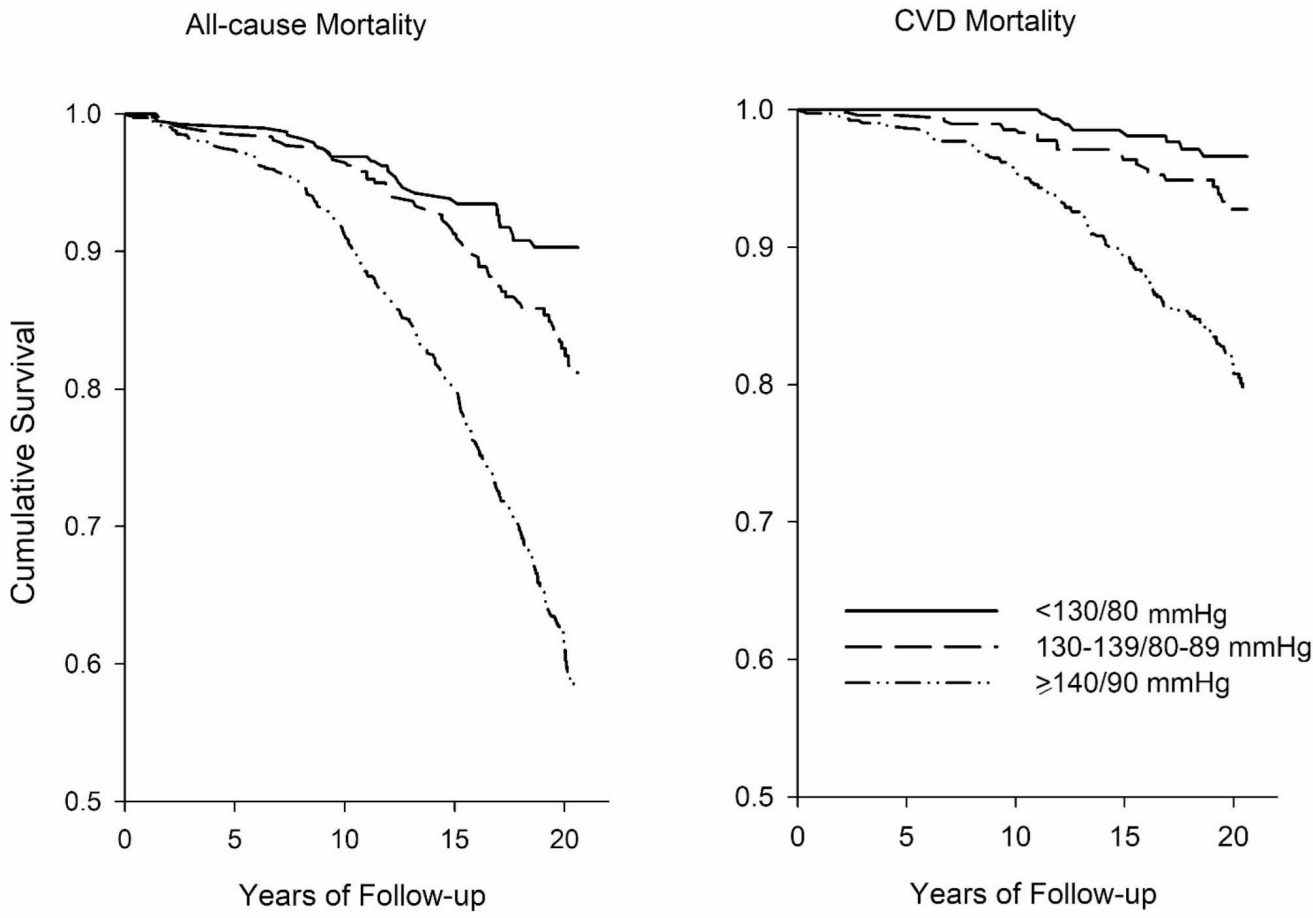
The effect BP categories on the incidence of all-cause and cardiovascular mortality among Chinese adults. Rates per 1,000 person-years were calculated in these three groups

During a median follow-up of 16.5 years, there were 397 deaths from all causes and 166 deaths from cardiovascular causes among the 2,004 subjects, with a total of 29,395 person-years. The corresponding incidence of all-cause mortality per 1,000 person-years for the BP < 130/80 mmHg, 130–139/80–89 mmHg, and ≥ 140/90 mmHg or treated groups were 4.5, 7.7 and 19.9, respectively, and of cardiovascular mortality were 1.3, 2.9 and 8.7, respectively (Fig. 1).

**Fig. 2:**
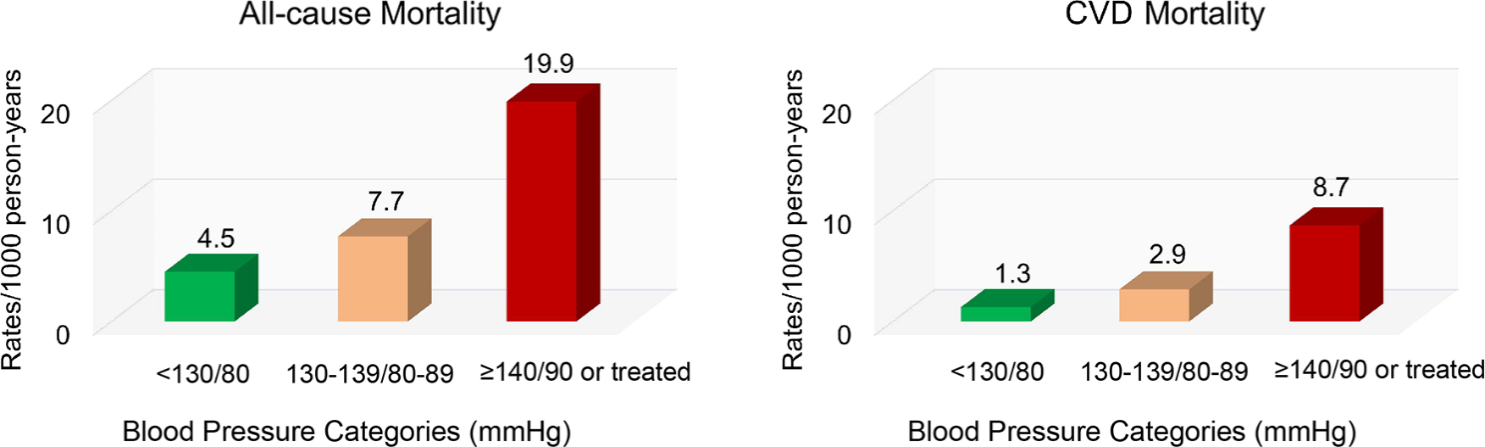
Long-term effects of blood pressure 130–139/80–89 mmHg on all-cause and cardiovascular mortality among Chinese adults

Using BP < 130/80 mmHg as the reference, the group with BP 130–139/80–89 mmHg showed significantly higher risks of all-cause and cardiovascular mortality in the crude model, but not in the adjusted model. After adjusting for age, sex, BMI, and other factors, multiple Cox regression analysis showed that BP ≥ 140/90 mmHg or treated was significantly associated with higher risks of all-cause mortality (HR 2.45 [95%CI 1.65–3.63]) and cardiovascular mortality (3.40 [1.65-7.00]) (Table 2). Figure 2 shows the Kaplan-Meier survival curve for all-cause and cardiovascular mortality according to BP category at baseline. The cumulative survival rates for all-cause and cardiovascular mortality were significantly lower in participants with BP ≥ 140/90 mmHg or treated.

**Table 2:** Multivariate-Adjusted Hazard Ratios for the risks of all-cause and CVD mortality according to the BP categories at baseline

	N/Total	Rate/1000 person-years	Crude HR (95% CI)	Adjusted HR (95% CI)	
				Model 1	Model 2
**All-cause mortality**					
**< 130/80**	28/407	4.5	1	1	1
**130–139/80–89**	58/520	7.7	1.78 (1.14–2.80) *	1.55 (0.99–2.44)	**1.54 (0.98–2.43)**
**≥ 140/90 or treated**	311/1077	19.9	4.59 (3.12–6.75) †	2.38 (1.61–3.51) †	**2.45 (1.65–3.63)†**
**CVD mortality**					
**< 130/80**	8/407	1.3	1	1	1
**130–139/80–89**	22/520	2.9	2.36 (1.05–5.31) *	2.13 (0.95–4.79)	**2.20 (0.98–4.99)**
**≥ 140/90 or treated**	136/1077	8.7	6.99 (3.43–14.3) †	3.27 (1.60–6.68) †	3.35 (1.63–6.91)† **3.40 (1.65-7.00)**†

Model 1 adjusted for age and sex. Model 2 adjusted for age, sex, BMI, family history of diabetes, history of CVD, physical activity, and cigarette smoking and alcohol status. *P < 0.05; †*P* < 0.01

### BP categories combined with blood glucose categories and all-cause and cardiovascular mortality

**Fig. 3:**
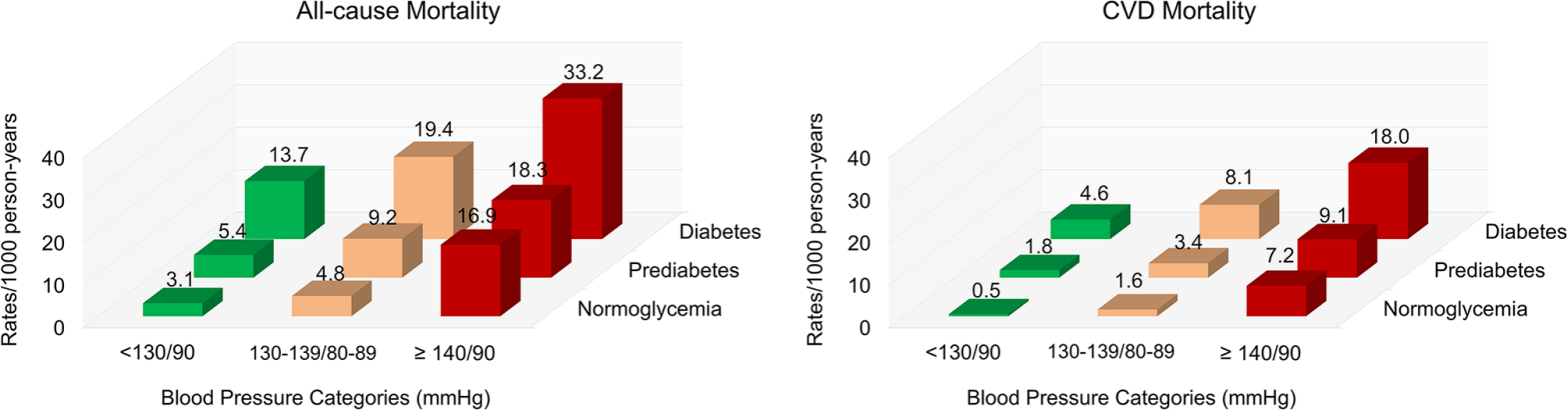
The effect of various blood glucose categories and BP categories combined on the incidence of all-cause and cardiovascular mortality among Chinese adults. Rates per 1,000 person-years were calculated in these nine groups

We assessed the risk for all-cause and cardiovascular mortality among participants stratified according to three BP categories (< 130/80 mmHg, 130–139/ 80–89 mmHg, and ≥ 140/90 mmHg or treated) and three blood glucose categories (normoglycemia, prediabetes, and diabetes) combined. Table 3 shows the adjusted HRs and 95% CIs for all-cause and cardiovascular mortality according to the various BP and blood glucose categories combined, using BP < 130/80 mmHg and normoglycemia as the reference. As expected, the coexistence of BP ≥ 140/90 mmHg or treated and diabetes were associated with the most significant increases in the all-cause mortality (HR 4.44 [95% CI 2.42–8.16]) and cardiovascular mortality (HR 11.8 [95% CI 2.86–48.7]). When compared with the reference group, BP of 130–139/80–89 mmHg combined with diabetes increased the all-cause mortality by **3.3** times and cardiovascular mortality by **9.6** times. BP ≥ 140/90 mmHg or treated and prediabetes in the same individual considerably increased the risk for all-cause mortality (adjusted HR 2.82 [1.54–5.16]) and cardiovascular mortality (adjusted 5.64 [1.36–23.4], respectively. However, BP of 130–139/80–89 mmHg combined with prediabetes was not associated with an elevated risk of all-cause and cardiovascular mortality in the crude and adjusted models. Figure 3 presents the effect of various categories of BP and glucose metabolism categories combined on the prevalence of all-cause and cardiovascular mortality among Chinese adults. Rates per 1,000 person-years were calculated in these nine groups.

**Table 3:** Multivariate-Adjusted Hazard Ratios for the risks of All-cause deaths according to BP categories and blood glucose categories at baseline

BP category (mm Hg)	All-cause Mortality				CVD mortality		
	n/total	Rate/1000 person-years	Adjusted HR (95% CI)*		n/total	Rate/1000 person-years	Adjusted HR (95% CI)*
**Normoglycemia**							
**< 130/80**	12/256	3.1	1		2/256	0.5	1
**130–139/80–89**	24/307	5.4	**1.52 (0.76–3.04)**		8/307	1.8	3.12 (0.66–14.8)
**≥ 140/90 or treated**	87/407	13.7	**2.57 (1.40–4.72)**		29/407	4.6	4.78 (1.14–20.2)*
**Prediabetes**							
**< 130/80**	9/124	4.8	**1.17 (0.49–2.78)**		3/124	1.6	**2.10 (0.35–12.6)**
**130–139/80–89**	22/167	9.2	**2.05 (1.01–4.17)**		8/167	3.4	**4.33 (0.92–20.5)**
**≥ 140/90 or treated**	119/434	19.4	**2.82 (1.54–5.16)†**		50/434	8.1	5.64 (1.36–23.4)*
**Diabetes**							
**< 130/80**	7/27	16.9	2.75 (1.08–7.01)*		3/27	7.2	**5.53(0.92–33.4)**
**130–139/80–89**	12/46	18.3	**3.30 (1.48–7.38)†**		6/46	9.1	**9.60 (1.93–47.7)†**
**≥ 140/90 or treated**	105/236	33.2	**4.44 (2.42–8.16)†**		57/236	18.0	**11.8 (2.86–48.7)†**

*Adjusted for age, sex, BMI, family history of diabetes, history of CVD, physical activity, and cigarette smoking and alcohol status. *P < 0.05; †*P* < 0.01

### Sensitivity analysis of the risks of all-cause and cardiovascular mortality for 130–139/80–89 mmHg

Sensitivity analysis was conducted to explore the impact of different baseline characteristics of the study population on the risk of all-cause and cardiovascular mortality for BP levels of 130–139/80–89 mmHg compared to BP levels below 130/80 mmHg. No significant differences in predictive values were observed across groups based on age, sex, BMI, smoking, drinking, and baseline 10-year ASCVD risk of ≥ 10% or not (Table 4).

**Table 4:** Adjusted Sensitivity Analyses on the Risk of All-cause deaths for BP 130–139/80–89 mm Hg as compared with that of < 130/80 mmHg

		N (case/total)	130–139/80–89 mm Hg vs. <130/80 mmHg			
			Rate / 1000 PYs	Hazard ratio (95% CI)*	*P*	*P*_int_
All-cause mortality						
**Sex**	Women	25/306	5.5	1.26 (0.66–2.42)	0.48	0.33
	Men	33/214	11.1	1.84 (0.94–3.60)	0.07	
Age	≥ 50 years	54/301	12.1	1.39 (0.84–2.30)	0.20	0.45
	< 50 years	4/219	1.3	0.89 (0.24–3.36)	0.87	
Body mass index	≥ 24.0 kg/m^2^	33/282	8.0	1.90 (0.92–3.94)	0.08	0.22
	< 24.0 kg/m^2^	25/238	7.3	1.15 (0.62–2.13)	0.65	
Current smoking in men	Yes	20/129	11.1	1.12 (0.49–2.56)	0.79	0.23
	No	13/85	11.3	3.19 (1.07–9.51)	0.001	
Alcohol intake in men	Yes	7/64	8.1	1.45 (0.35-6.00)	0.61	0.56
	No	26/150	12.5	2.30 (1.04–5.06)	0.04	
ASCVD risk score	≥ 10%	8/30	34.3	1.93 (0.49–7.54)	0.94	0.65
	< 10%	31/490	4.8	1.22 (0.67–2.21)	0.52	
CVD mortality						
**Sex**	Women	10/306	2.2	1.49 (0.51–4.38)	0.47	0.21
	Men	12/214	4.1	3.52 (0.96–12.9)	0.06	
Age	≥ 50 years	21/301	4.7	2.01 (0.85–4.79)	0.11	0.12
	< 50 years	1/219	0.3	0.65 (0.04–10.5)	0.76	
Body mass index	≥ 24.0 kg/m^2^	11/282	2.7	1.74 (0.54–5.64)	0.35	0.45
	< 24.0 kg/m^2^	11/238	3.2	2.76 (0.86–8.85)	0.09	
Current smoking in men	Yes	4/129	2.2	1.07 (0.18-6.00)	0.94	0.08
	No	8/85	7.0	12.8 (1.44–114.0)	0.02	
Alcohol intake in men	Yes	1/64	1.2			
	No	11/150	5.3	4.57 (0.98–21.2)	0.06	
ASCVD risk score	≥ 10%	3/30	17.2	1.65 (0.23–11.8)	0.94	0.46
	< 10%	19/490	1.6	1.65 (0.51–5.29)	0.40	

*Adjusted for age, sex, body mass index, smoking and drinking, use of antihypertensive drugs, mean arterial pressure, family history of diabetes, and serum cholesterol, as appropriate

## Discussion

In this comprehensive study, we thoroughly investigated the potential risks of all-cause and cardiovascular mortality among a Chinese population-based cohort with a BP range of 130–139/80–89 mmHg. Our groundbreaking findings implied that individuals falling within this BP range face a notably higher risk of both all-cause and cardiovascular mortality when accompanied by a diagnosis of diabetes. Importantly, these risks persist over an extended follow-up period of more than 20 years, emphasizing the long-term implications of elevated BP levels in this specific population.

### BP targets and CVD risk

BP of 130–139/80–89 mmHg has different definitions in various guidelines. These definitions are primarily based on the differing understanding of the long-term effects of BP in this range on outcomes, such as cardiovascular events and mortality. Several individual studies and meta-analyses of observational data have reported a progressively higher risk of CVD from < 120/80 to 130–139/80–89 mmHg [24–26]. In many of these meta-analyses, the hazard ratios for coronary heart disease and stroke ranged from 1.1 to 1.5 for BP of 120–129/80–84 mmHg, and from 1.5 to 2.0 for BP of 130–139/85–89 mmHg compared to < 120/80 mmHg. However, there is still controversy regarding the association of BP of 130–139/80–89 mmHg with all-cause mortality. A meta-analysis of prospective cohort studies showed that BP of 120–139/80–89 mmHg increased the risk of CVD by 55% and cardiovascular mortality by 17%, but not increase the risk of all-cause mortality [26]. Another pooled analysis of three prospective Chinese cohorts with a total follow-up of 1,718,089 person-years showed that BP of 130–139/80–89 mmHg increased the risk of cardiovascular mortality by 40%, but not of all-cause mortality (HR 1.04, 95% CI 0.89–1.21), compared to BP < 120/80 mm Hg [27]. In our study, we found that BP of 130–139/80–89 mmHg was not significantly with all-cause and cardiovascular mortality in Chinese adults, whether in crude models or when adjusted for age, sex, BMI, family history of diabetes, physical activity, and cigarette smoking and alcohol status. These finding are consistent with our previous finding on CVD events [17].

### BP target in patients with diabetes

There has been extensive discussion over the past decade about individualized blood pressure targets for patients with diabetes and hypertension. In fact, hypertension and optimal blood pressure targets are more complex in patients with diabetes than in those with hypertension alone. Hypertension and impaired glucose metabolism have distinct genomic characteristics, emphasizing the need for studies on the impact of genomic features on pure and combined forms of the diseases [28]. In a meta-analysis of randomized controlled trials involving patients with diabetes or prediabetes, lowering systolic blood pressure to ≤ 135 mmHg was associated with a 10% reduction in all-cause mortality compared to lower intensity control, while more intensive blood pressure control (≤ 130 mmHg) was more strongly associated with a reduction in stroke risk but did not lower other events [29]. For years, guidelines have recommended keeping patients’ blood pressure controlled below 130/80 mm Hg [30, 31]. The basis for these guidelines is the UK Prospective Diabetes Study 36^10^, UK Prospective Diabetes Study [32], the Hypertension Optimal Treatment (HOT) trial [33], the Appropriate Blood Pressure Control in Diabetes (ABCD) trial [34], and the Action in Diabetes and Vascular Disease: Preterax and Diamicron MR Controlled Evaluation (ADVANCE) study [35]. However, after the publication of the Action to Control Cardiovascular Risk in Diabetes (ACCORD)-BP study [36], the treatment target for patients with diabetes was changed in 2013 to a blood pressure lower than 140/90 mm Hg, leading to updates in multiple hypertension guidelines [30, 31] Subsequent systematic reviews concluded that treating individuals with blood pressure already below 140 mm Hg is associated with a reduced risk of stroke and albuminuria [37], thus questioning the relaxation of guidelines. In an observational study conducted in Japan, it was found that a home SBP of at least 125 mmHg in the morning was an independent predictor of cardiovascular events in individuals with diabetes. It has been proposed that diabetic East Asians should aim for a home morning SBP below 125 mmHg [38]. The latest hypertension guidelines recommend controlling blood pressure targets below 130/80 mm Hg for patients with diabetes [6, 7].

To determine the optimal BP threshold among patients with diabetes and hypertension, it is crucial to consider its influence on long-term outcomes. In this study, which spanned 21 years and involved a population-based prospective cohort, we examined the incidence of all-cause and cardiovascular mortality among individuals with a BP of 130–139/80–89 mmHg and either diabetes or prediabetes. After assessing the association between BP categories and the risk of all-cause mortality and cardiovascular mortality, we further analyzed the risk of mortality by combining BP categories with blood glucose categories. As expected, we found that individuals with a BP ≥ 140/90 mmHg (or treated) and diabetes had the highest incidence of all-cause mortality (HR 4.51) and cardiovascular mortality (HR 12.0). Of note, individuals with a BP of 130–139/80–89 mmHg combined with diabetes had a 3.26 times higher risk of all-cause mortality and a 9.53 times higher risk of cardiovascular mortality. Interestingly, a BP of 130–139/80–89 mmHg combined with prediabetes did not show an increased risk of all-cause and cardiovascular mortality in both crude and adjusted models. To our knowledge, this study provides the first data on the occurrence of all-cause and cardiovascular mortality among Chinese adults with a BP of 130–139/80–89 mmHg and either diabetes or prediabetes in a population-based prospective cohort.

### BP target in individuals with prediabetes

Prediabetes is an intermediate glycemic state between normal glycemia and diabetes, and it affects about half of the population in China. There is inconsistent association between prediabetes and CVD disease, as well as cardiovascular and all-cause mortality [39, 40]. The association between prediabetes and CVD risk may be affected by hypertension status. Two earlier studies conducted in China showed that individuals with prediabetes and hypertension had a higher risk of CVD events compared to those without prediabetes or hypertension [18, 41]. However, a recent study conducted on 3,313 black adults in the Jackson Heart Study (JHS) without diabetes or a history of CVD at baseline found that prediabetes was not associated with an increased risk of cardiovascular or all-cause mortality, regardless of hypertension status [42]. The present study shows that individuals with prediabetes combined with a BP of ≥ 140/90 mmHg or who are being treated for hypertension indeed have a higher risk of cardiovascular and all-cause mortality.

### Strengths and limitations

Our study should be interpreted within the context of its strengths and limitations. One of the strengths of our study is a population-based prospective cohort with the extensive follow-up period of 21 years, allowing us to track outcomes over a long period of time. However, it is important to acknowledge the limitations of our analyses as well. Firstly, not all participants underwent a baseline HbA1c test in 2002, which may have resulted in some cases of diabetes being missed if they were only detected by HbA1c. This potential oversight could have affected the overall picture of diabetes prevalence in our study. Nevertheless, it is worth noting that the clinical diagnosis of both prediabetes and diabetes was primarily based on FPG and 2hPG levels. HbA1c was not included in the diagnostic criteria until 2010, so our findings are based on the established criteria at the time of the study. Secondly, our study used office BP measurements instead of 24-hour ambulatory BP monitoring. It has been shown that 24-h ambulatory systolic BP, but not office BP, is independently associated with cardiac remodeling, coronary microvascular dysfunction, and diastolic dysfunction among asymptomatic individuals with Diabetes Mellitus [43]. Thirdly, our study has a relatively small sample size and includes participants who are relatively young. Despite a follow-up period of over 20 years, the number of deaths in certain subgroups is still low. This limitation may result in the analysis lacking sufficient statistical power for these subgroups. Given the relatively small sample size in this study, we are unable to distinguish statistically whether the observed risk is primarily attributed to blood pressure or diabetes. However, we can observe the additive effect of both blood pressure and diabetes status. Forth, since serum creatinine and urinary albumin levels were not measured at baseline, it was not possible to evaluate the related information of chronic kidney disease in participants with a BP of 130–139/80–89 mmHg. Finally, it is important to note that this study is solely observational in nature. Therefore, further research in the form of randomized controlled clinical trials is needed to evaluate the potential benefits derived from antihypertensive treatment among this specific patient population.

## Conclusions

In conclusion, our findings of this study suggest that individuals with diabetes and a BP of 130–139/80–89 mmHg may face a significantly higher risk of all-cause and cardiovascular mortality over an extended follow-up period of more than 20 years. This highlights the importance of maintaining a target BP level below 130/80 mmHg for individuals with diabetes.

## Data Availability

All data generated or analyzed during this study are available in this published article.
